# Oxygen functional groups improve the energy storage performances of graphene electrochemical supercapacitors†

**DOI:** 10.1039/c7ra12425b

**Published:** 2018-01-12

**Authors:** Hailiang Cao, Xing Peng, Min Zhao, Peizhi Liu, Bingshe Xu, Junjie Guo

**Affiliations:** Key Laboratory of Interface Science and Engineering in Advanced Materials, Ministry of Education, Research Center of Advanced Materials Science and Technology, Taiyuan University of Technology Taiyuan 030024 China caohltyut@sohu.com guojunjie@tyut.edu.cn; Key Laboratory of Graphene Technologies and Applications of Zhejiang Province, Advanced Li-ion Battery Engineering Lab, Ningbo Institute of Materials Technology and Engineering (NIMTE), Chinese Academy of Sciences Ningbo 315201 China

## Abstract

Graphene is a promising electrode material for supercapacitors due to its superior physical and chemical properties, but the influence of its oxygen functional groups on capacitive performance still remains somewhat uncertain. In this work, graphene sheets with different oxygen content have been prepared through thermal reduction in argon. Furthermore, oxidation and pore-forming treatment of graphene annealed at 800 °C are also performed to explore the important effect of oxygen functional groups. The effects of disorder degree, surface area and oxygen functional groups on the specific capacitance were explored systematically. The content and species of oxygen functional groups are found to be significant factors influencing the electrochemical supercapacitor performance of graphene electrodes. The specific capacitances of graphene annealed at 200, 400 and 800 °C are 201, 153 and 34 F g^−1^, respectively. However, the specific capacitance of graphene reduced at 800 °C can be increased to 137 F g^−1^ after nitric acid oxidation treatment, and is only 39 F g^−1^ after pore forming on graphene surface, demonstrating that the oxygen functional groups can improve the capacitive performances of graphene electrochemical supercapacitors.

## Abstract 2

The content and species of oxygen functional groups are significant factors influencing the electrochemical supercapacitor performance of graphene electrodes.

## Graphical abstract

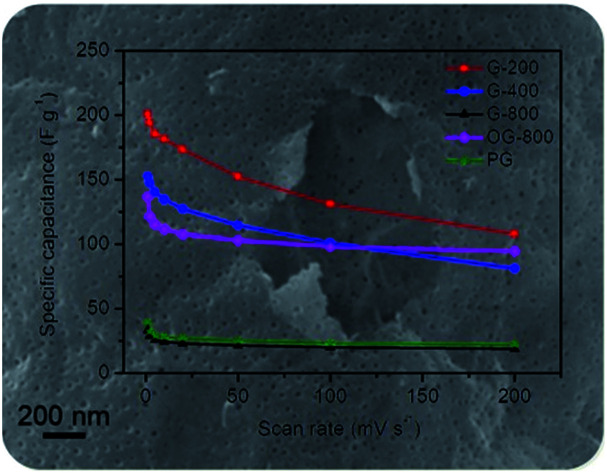

## Introduction

Supercapacitors, one of the most promising energy-storage devices with high power density and long cycle life, have drawn considerable attention due to their wide potential in hybrid power sources, such as electrical vehicles and uninterruptible power supplies.^1–3^ The electrode material is the central component of a supercapacitor and drastically dictates its ultimate performance. Commonly, porous carbon (activated carbon, carbon aerogel, carbon nanotubes, and template carbon, *etc.*) is used as the electrode material for supercapacitors.^4–8^ Graphene, a monolayer of sp^2^ hybridized carbon atoms in honeycomb structure, has recently attracted extensive interests as a promising supercapacitor electrode material owing to its large theoretical specific surface area, high intrinsic electrical conductivity, outstanding mechanical flexibility and high chemical stability.^9–11^

Since Ruoff *et al.*^12^ and Vivekchand *et al.*^13^ pioneered supercapacitors based on graphene by chemical and thermal method, respectively, there have been numerous reports on the application of graphene-based electrode materials as supercapacitors.^11,14–18^ Generally, chemical attribute is also a critical factor for the capacitive performance of porous carbon besides specific surface area, electrical conductivity and pore geometry.^19^ Furthermore, surface functional groups including nitrogen, oxygen and phosphorous can considerably increase the capacitance through pseudo-capacitance effects, as well as improving the wettability of porous carbon with electrolytes.^20–25^ It have been widely demonstrated that the doping heteroatoms (such as N, B or P), which contribute to the pseudo-capacitance, is an effective approach to improve the capacitive performance of graphene.^26–29^ Nevertheless, the oxygen functional groups also play significant role in affecting the capacitive property of graphene as supercapacitors electrode material, to which little attention have been payed.^30–32^ In most cases for graphene with high specific capacitance value, C/O ratios are relatively low depending on the synthetic rout.^11^ In addition, Xu *et al.* reported that graphene oxide (GO) shows a higher capacitance than graphene because its abundant oxygen-containing functional groups provide additional pseudo-capacitance.^19^ However, Banks and his coworkers demonstrated the inverse results, in which the specific capacitance increases when the oxygenated species reduces.^30^ While only the electrochemical performance of graphene and GO were investigated in the two works mentioned above. Moreover, in most researches, graphene electrode materials prepared by reducing GO inevitably possess a certain amount of oxygen functional groups.^33–37^ Thus, it should never be neglected that the effect of oxygen functional groups to capacitive behavior of graphene electrochemical supercapacitor. Consequently, the systematic investigation is necessary and urgent to clarify the contradictory understanding.

In this work, we present a systematic investigation about influence of oxygen functional groups on capacitive properties of graphene as the electrode material for supercapacitors. Graphene with different oxygen content were synthesized by changing thermal reduction temperature. The effects of structural parameters such as disorder degree, surface area and oxygen content on specific capacitance were studied in detail. Our results indicate that oxygen functional groups in graphene are the critical factor improving their electrochemical capacitor performance. The specific capacitance of graphene annealed at 800 °C can be raised from 34 to 137 F g^−1^ after oxidation treatment. This work probably provides a new insight for designing and synthesizing graphene-based materials of supercapacitors.

## Experimental

### Materials preparation

GO was synthesized from natural graphite flakes using a modified Hummers method.^38^ The final suspension of GO was frozen in a fridge (−18 °C), and then transferred into a freeze drying equipment and evaporated in vacuum at the temperatures below 0 °C for 2 days to obtain GO powders. Graphene was prepared by thermally reducing GO powders in a simple horizontal tube furnace in argon atmosphere with heating rate of 10 °C min^−1^. The annealing temperature was varied from 200 to 800 °C for 1 h to control the deoxygenation degrees of graphene. The obtained samples were denoted as G-200, G-400 and G-800, respectively.

G-800 was oxidized by nitric acid to study the importance of oxygen functional groups. Typically, 0.2 g G-800 and 60 mL nitric acid were mixed at room temperature and subsequently stirred for 12 h and then washed and filtered. After drying under vacuum, oxidized G-800 (OG-800) were obtained.

Porous graphene (PG) was prepared by a metal etching method as reported in our previous work,^39^ and the detailed synthetic procedure is as following. GO suspension mixed homogeneously with cobalt acetate solution through severe stirring. The mass ratio of GO/Co was 10/1. After sonication, the final solution was frozen, transferred into freeze drying equipment. The dry mixture powder was heated at 800 °C for 1 hour in a tube furnace under a flowing Ar gas. Finally, the black powder was treated by diluted hydrochloric acid, followed by washing with water. The resulting powder was dried under vacuum to obtain PG.

### Sample characterization

SEM images were acquired using a scanning electron microscopy (LYRA3 XMH, Tescan). X-ray photoelectron spectra (XPS) were carried out by an AXIS ULTARDLD spectroscopy from Kratos. Nitrogen adsorption/desorption measurements were recorded at 77 K in Micromeritics ASAP 2020M instrument. Raman spectra analysis was conducted by a Renishaw inVia Reflex Raman Spectrometer, with a 532 nm-wavelength laser. FT-IR spectra were collected using a Bruker Tencor II instrument. Contact angle meter SL200B (Solo Tech. Co., Ltd.) was employed to measure the contact angle of the samples.

### Electrochemical measurements

The electrochemical properties of as-prepared samples for supercapacitors were investigated using CR2032 coin cells at room temperature. A slurry was prepared first by mixing the active materials, polyvinylidene fluoride and Super P with a weight ratio of 8 : 1 : 1 in *N*-methylpyrrolidone solution. Then the slurry was casted on nickel foam and dried at 100 °C for 12 h in vacuum. Finally, the coin cell, which was a symmetrical two-electrode unit cell, was assembled using a glass fiber filter paper as the separator. Several drops of 5 M KOH as the electrolytes were added before the cell was pressed to seal for measurement. The cyclic voltammogram (CV) and galvanostatic charge/discharge tests were carried out using CHI 760E. The cycling performance was tested through galvanostatic charge/discharge using a LAND CT2001A battery test system. The electrochemical impedance spectroscopy (EIS) was characterized by using a Princeton Verstat3 electrochemical workstation. The frequency range for impedance spectra was from 100 kHz to 0.01 Hz with 10 mV voltage amplitude.

To quantitatively compare the capacitance performance of these electrode materials, the values of specific capacitance *C*_s_ were calculated according to the following equations:
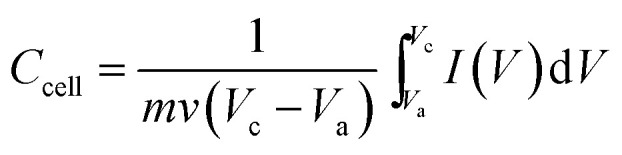
*C*_s_ = 4*C*_cell_where *C*_cell_ is the specific capacitance of the coin cell, *m* is the total mass of two electrodes, *v* is the scan rate, (*V*_c_ − *V*_a_) represents the sweep potential range, and *I*(*V*) denotes the response current.

## Results and discussion

**Fig. 1:**
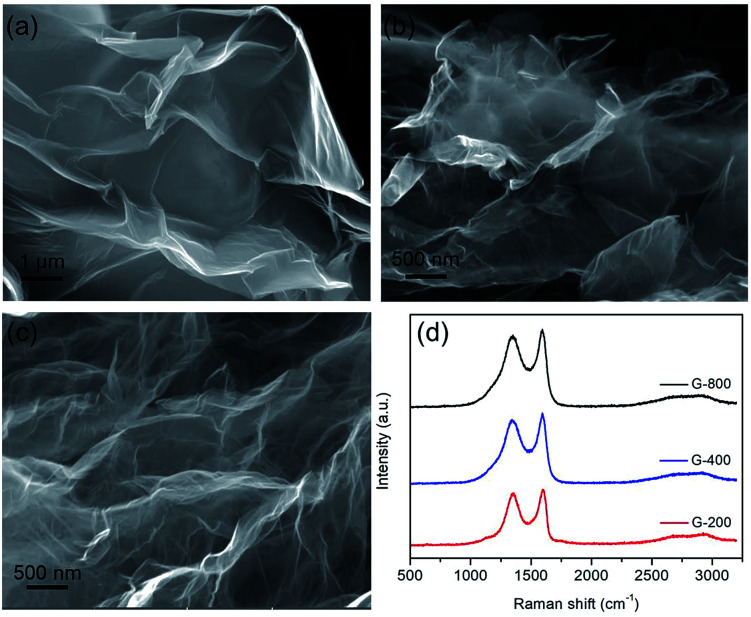
The microstructures of graphene with different reduction levels. SEM images of G-200 (a), G-400 (b) and G-800 (c), and their corresponding Raman spectra (d).

Graphene nanosheets with different content of oxygen functional groups were prepared by reducing GO at different pyrolytic temperatures from 200 to 800 °C, which were denoted as G-200, G-400 and G-800. Their morphology and nanostructure were characterized by SEM observation. Typically crumpled structures are observed for all samples with a lightly trend toward higher level of wrinkling when annealing temperature increased (Fig. 1a–c).^27^ Moreover, the graphene nanosheets of the three samples are transparent due to their very thin thickness. In addition, Raman spectra of these samples are depicted in Fig. 1d. Two typical peaks line around 1345 cm^−1^ (D band) and 1595 cm^−1^ (G band) are resolved for all samples, which assigned to the E_2g_ phonon of C sp^2^ atoms and breathing mode of *κ*-point phonon of *A*_1g_ symmetry, respectively.^31,40,41^ The intensity ratios of the D to G bands (*I*_D_/*I*_G_) are listed in Table S1,† reflecting the structural changes. The *I*_D_/*I*_G_ ratios of G-200, G-400 and G-800 are 0.96, 0.94 and 0.93, respectively. The decreasing *I*_D_/*I*_G_ ratio with increasing the pyrolytic temperature indicates the reduction of disorder degree and the increase in size of sp^2^ clusters.^32^ Furthermore, the BET surface area of G-200, G-400 and G-800 are tested to be 248, 302 and 251 m^2^ g^−1^, respectively. The much lower value than the theoretical limit of pristine graphene is probably caused by partial overlap and coalescence of graphene nanosheets during reduction process.^42^

**Fig. 2:**
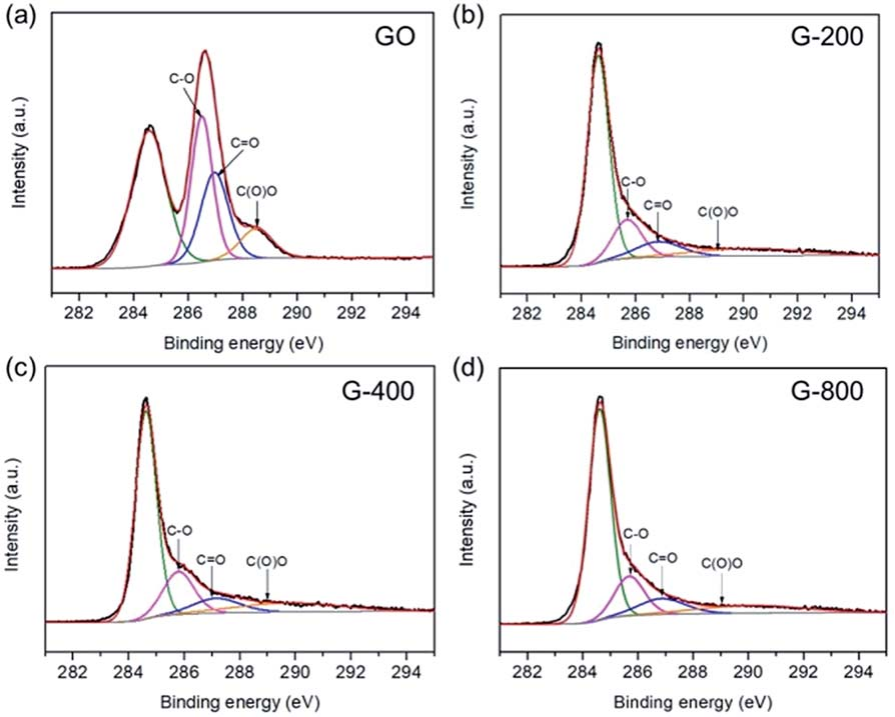
The C 1s XPS spectra of GO (a), G-200 (b), G-400 (c) and G-800 (d).

The as-prepared samples were characterized by FTIR spectra. Fig. S1† shows the FTIR spectra of GO, G-200, G-400 and G-800, respectively. The spectra of these samples in our experiment are similar to the results reported by previous researches.^43,44^ The spectrum of GO indicates the presence of C=O (1732 cm^−1^), aromatic C=C (1623 cm^−1^), carboxy C–O (1390 cm^−1^), and epoxy or alkoxyl C–O (1050 cm^−1^). While the band at *ca.* 3423 cm^−1^ could be due to the O–H stretching mode of intercalated water. After thermal treatment, the peaks for oxygen functional groups were reduced significantly, and the peak at 1390 cm^−1^ for carboxy C–O was nearly entirely removed. After annealed at 800 °C, the carboxyl groups are considerably decreased. The surface chemistry of GO before and after thermal reduction were further characterized using X-ray photoelectron spectroscopy (XPS). The XPS spectra of GO, G-200, G-400 and G-800 over a wide range of binding energy (0–1000 eV) are shown in Fig. S2.† It's obvious that the oxygen content of the graphene decreases with increasing the annealing temperature. The specific carbon and oxygen content of these samples were summarized in Table S1.† The C/O atomic ratios measured by XPS survey spectra are calculated to be 4.1, 8.3 and 13.5 for G-200, G-400 and G-800, respectively, confirming the thermal decomposition of the functional groups. The bonding configurations of C atoms in these samples were investigated by high resolution XPS and the results are displayed in Fig. 2. The C 1s peak for all samples can be resolved into four components centered at 284.6, ∼285.6, ∼286.9 and ∼289.5 eV, which can be attributed to the sp^2^ C–C, C–OH, C=O and O=C–OH groups, respectively.^40,45,46^ It should be mentioned that these graphene samples also possess an epoxy group (C–O–C), which have a C 1s binding energy similar to C–OH.^40,46^ The area percentages of each peak, which corresponds to the content of these four C species are summarized in Table S1.† With the rise of annealing temperature, the content of sp^2^ C–C increases, which matches well with the Raman results. In a word, there are obvious differences among the content and species of oxygen functional groups of the three grapheme samples. Thus the electrochemical properties of these graphene electrode materials may be different.

**Fig. 3:**
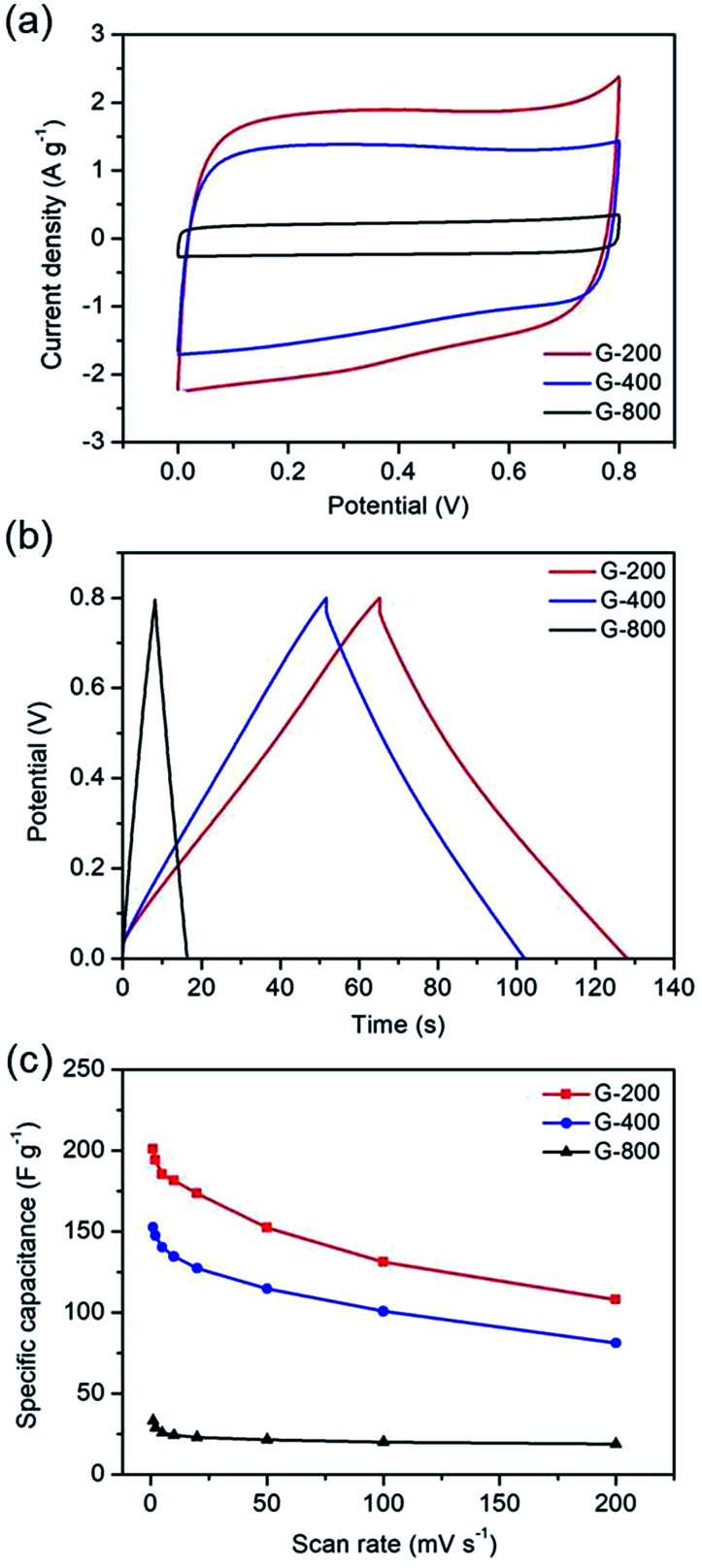
The electrochemical capacitive behavior of graphene with different oxygen content. (a) Cyclic voltammetry at a scan rate of 20 mV s^−1^. (b) Galvanostatic charge–discharge curves at a current density of 1 A g^−1^. (c) Specific capacitance at different scan rates.

The electrochemical performances of the as-prepared samples were measured in a symmetrical two-electrode cell, which can provide the most accurate measurement of material performance for the supercapacitor.^47^Fig. 3a exhibits the CV curves of G-200, G-400 and G-800 at 20 mV s^−1^ scan rate in 5 M KOH electrolyte between 0 and 0.8 V. The CV curves of G-200 and G-400 show a box-like shape with a well-broadened peak at 0–0.6 V, inferring the presence of pseudo-capacitance. However, the CV curves of G-800 shows a nearly rectangular shape, which is typical of a capacitance behavior. Moreover, the capacitive behavior difference among the three samples is confirmed by galvanostatic charge–discharge curves at a current density of 1 A g^−1^, as shown in Fig. 3b. The charge–discharge curve of G-800 shows an ideal linear shape, while a little deviation from the line at lower potentials can be observed for G-200 and G-400. The deviations of the CV and charge–discharge curves are related to quick faradaic reactions, due to the presence of additional oxygen containing groups at the surface of G-200 and G-400.^19,20^ In addition, the CV curves for these three samples at different scan rates ranging from 1 to 200 mV s^−1^ were measured (Fig. S3†).

Fig. 3c shows the specific capacitance of the three samples at different scan rates. The specific capacitances of G-200, G-400 and G-800 are 201, 153 and 34 F g^−1^, respectively, at a scan rate of 1 mV s^−1^. G-200 and G-400 show several-fold increased capacitances compared to that of G-800 case over all scan rates. At scan rate of 20 mV s^−1^, the specific capacitance of G-800 is only 23 F g^−1^. In contrast, the specific capacitance of G-400 and G-200 can reach up to 127 and 174 F g^−1^, respectively, six to eight times larger than the value of G-800. Even when the scan rate increase to 200 mV s^−1^, the capacitance of G-200 and G-400 can remain at 108 and 81 F g^−1^, respectively, implying a quick charge propagation capability of both double layer capacitance and pseudo-capacitance. It is observed that the specific capacitance of graphene samples decreases in the order of G-200 > G-400 > G-800, which is coincident with the sequence of the percentage of oxygen in three samples (G-200 > G-400 > G-800). The specific capacitance decreases when the oxygenated species reduces, demonstrating a remarkable contribution of oxygen-containing functional groups in raising the capacitance of graphene. The rate performances of these samples were also investigated by galvanostatic charge/discharge at different current densities (Fig. S4†). With an increase of the current density, the capacitances of the three samples decrease slowly, indicating the excellent rate capabilities. The specific capacitance of G-200 and G-400 remain 125 and 95 F g^−1^, respectively, even at 5 A g^−1^.

**Fig. 4:**
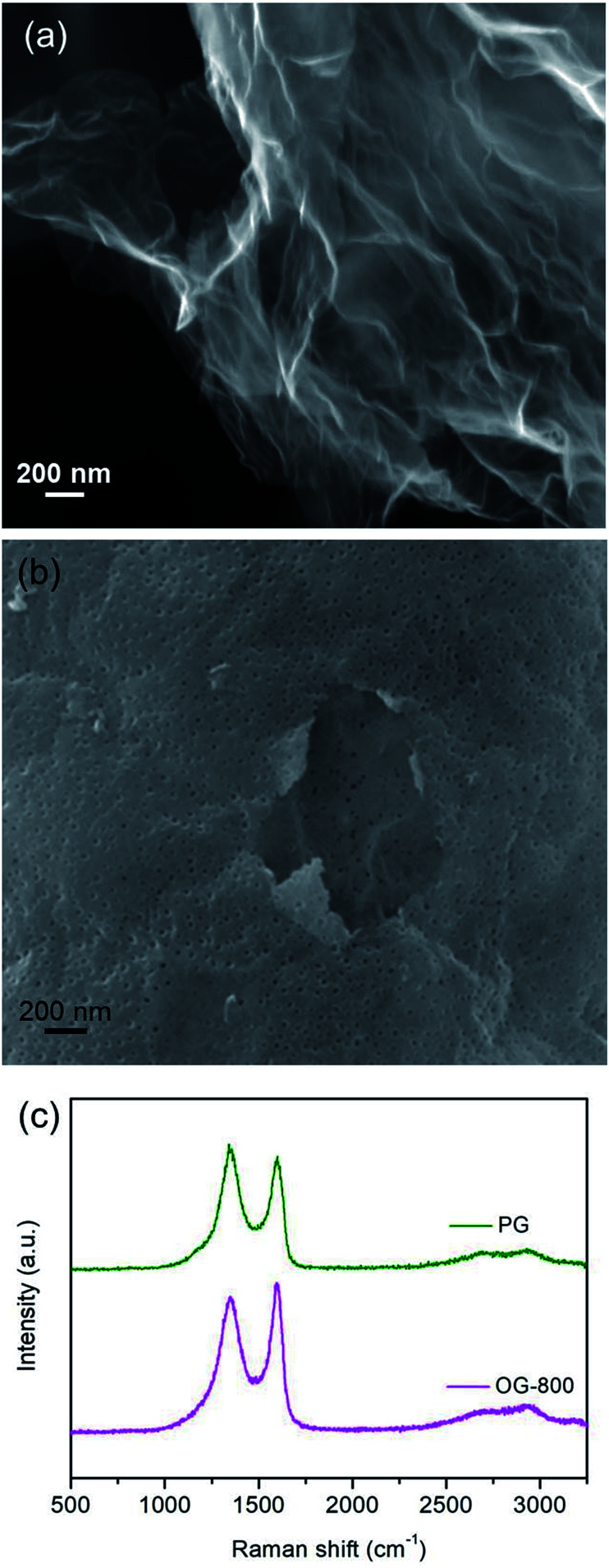
SEM images of (a) OG-800 and (b) PG. (c) Raman spectra of these samples.

In order to further demonstrate the effect of the oxygen-containing groups on energy storage performance of graphene electrode, two modified graphene samples were prepared. One is OG-800 obtained from oxidation treatment of G-800 using nitric acid. From Fig. 4a we can see that the morphology of G-800 almost unchanged after oxidation, remaining thin and transparent nanostructure. And the other one is PG synthesized through metal etching method. Metal nanoparticles can react with carbon atoms of graphene to form porous graphene at high temperatures, the formation mechanism of which was reported in our previous research.^39^ The cobalt metal was employed in this work to prepare the PG. After thermal treatment at 800 °C, many cobalt nanoparticles with the similar size of the pore distribute evenly on the surface of graphene, and some pores can be observed clearly beside the nanoparticles (Fig. S5†). Furthermore, the SEM image in Fig. 4b shows the typical morphology of PG. A significant amount of pores appeared on the intact graphene surface after removing the cobalt nanoparticles using hydrochloric acid. In addition, the Raman spectra also reflect the structural differences of the two samples (Fig. 4c). The *I*_D_/*I*_G_ ratios of PG and OG-800 are 1.04 and 0.92, respectively, indicating the existence of plenty defects in graphene sheets of PG. Moreover, the BET surface area of PG and OG-800 are 116 and 240 m^2^ g^−1^, respectively. The specific surface area of G-800 almost didn't change before and after oxidation treatment. While the specific surface area of PG reduced by half compared to that of G-800, which mainly due to the inevitable aggregation of graphene nanosheets during the process of preparation.^39^

**Fig. 5:**
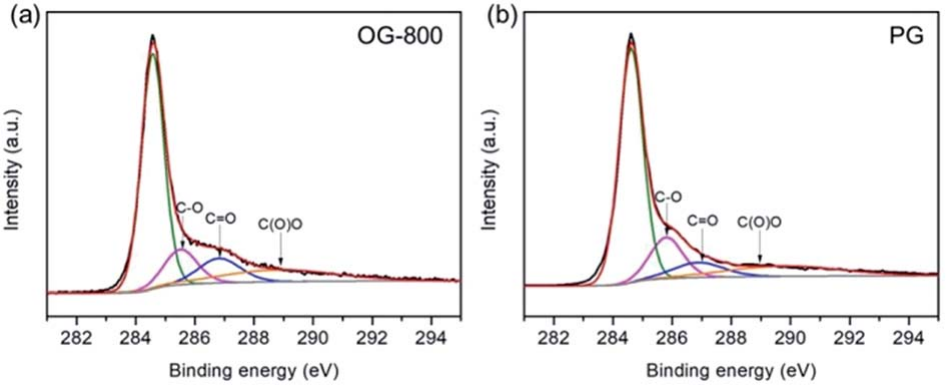
The C 1s XPS spectra of OG-800 (a) and PG (b).

XPS was applied to determine the chemical constituents of functional groups in PG and OG-800, as shown in Fig. S6.† The intensity of O 1s speak of OG-800 is higher than that of G-800, indicting higher oxygen-related contents. The C/O atomic ratios for PG and OG-800 were calculated to be 14.2 and 6.5, respectively. Moreover, as shown in Fig. S7,† the spectrum of OG-800 illustrates that the peaks for C=O and C–O increase obviously after oxidation treatment. Fig. S8† shows the wettability of the samples. The water contact angles of the G-200, G-400, G-800 and OG-800 are 94°, 126°, 137° and 108°, respectively. Clearly, the hydrophobicity of the samples increases with raising annealing temperature. However, the hydrophilicity of OG-800 was improved after oxidation treatment, which is helpful for increasing its electrochemical performance. In addition, Fig. 5 shows the curve fittings of high-resolution C 1s spectra of OG-800 and PG. The C 1s spectra also have been resolved into four individual component peaks. The quantitative peak analysis of C 1s peak region by calculating the integrated peak area was also summarized in Table S1.† The each peak area of C 1s peak of PG is very similar to that of G-800. However, the content of sp^2^ C–C of OG-800 still remain 60.03%, a slight drop compared to that of G-800.

**Fig. 6:**
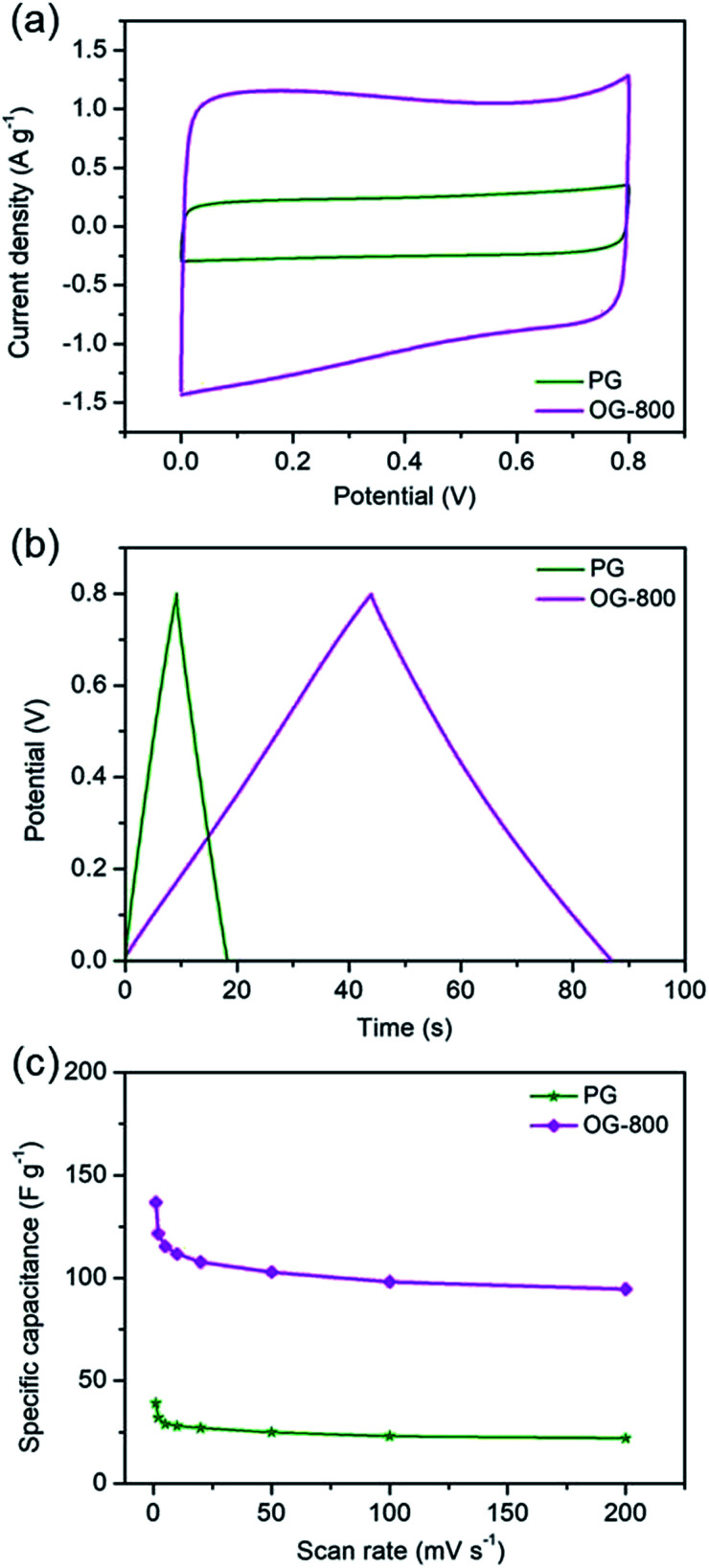
Electrochemical measurements of PG and OG-800. (a) Cyclic voltammograms at a scan rate of 20 mV s^−1^. (b) Charge–discharge curves at a current density of 1 A g^−1^. (c) Specific capacitance at different scan rates.

The electrochemical performances of PG and OG-800 were also measured, as shown in Fig. 6. Fig. 6a exhibits the CV curves of PG and OG-800 at 20 mV s^−1^ scan rate. The CV curve of OG-800 shows a box-like shape similar with that of G-200 and G-400, indicating the presence of pseudo-capacitance. However, the CV curve of PG is similar to that of G-800, revealing a typical capacitance behavior. Moreover, galvanostatic charge–discharge curves (Fig. 6b) also demonstrate the capacitive behavior difference among the two samples. The charge–discharge curve of PG shows an ideal linear shape, while a little deviation from the line at lower potentials can be observed for OG-800. Importantly, as we can see from Fig. 6a and b, the specific capacitance of OG-800 is obviously higher than that of PG. Furthermore, the CV curves for PG and OG-800 at different scan rates ranging from 1 to 200 mV s^−1^ were also measured (Fig. S9†). The shape of CV curves does not change markedly as the scan rate increased, indicating fast charge transfer within the two electrodes. Fig. 6c shows the rate performance of the two samples. It's worth noting that the specific capacitance of PG is only 27 F g^−1^ at 20 mV s^−1^, which is close to the value of G-800 (23 F g^−1^). In contrast, the specific capacitance of OG-800 is as high as 107 F g^−1^, approximate five times larger than the value of G-800, demonstrating that the oxygen functional groups can greatly improve the capacitive performance of graphene electrode materials. However, not all these groups are beneficial for reversible electrochemical redox reaction. Previous researches reveal that redox reaction between quinone and phenol groups contribute additional pseudocapacitance, while surface acidic functional groups (carboxyl-type) play negative role in supercapacitors.^31,48,49^ G-400 and OG-800 were chose for comparison because the oxygen contents of the two samples are relatively close. As shown in Table S1,† the specific capacitance of G-400 is 127 F g^−1^, which is higher than that of OG-800. Moreover, the C=O and COOH group contents of G-400 are 9.40% and 12.91%, respectively, which are lower than that of OG-800. However, the C–OH group content of G-400 is 17.89%, which is larger than that of OG-800 (13.17%). Therefore, we infer that the C–OH group is more contributive to the improvement of capacitive performance. The pseudo-capacitance of graphene probably comes from the electrochemical reactions, *e.g.* <svg xmlns="http://www.w3.org/2000/svg" version="1.0" width="10.400000pt" height="16.000000pt" viewBox="0 0 10.400000 16.000000" preserveAspectRatio="xMidYMid meet"><metadata> Created by potrace 1.16, written by Peter Selinger 2001-2019 </metadata><g transform="translate(1.000000,15.000000) scale(0.011667,-0.011667)" fill="currentColor" stroke="none"><path d="M80 1160 l0 -40 40 0 40 0 0 -40 0 -40 40 0 40 0 0 -40 0 -40 40 0 40 0 0 -40 0 -40 40 0 40 0 0 -40 0 -40 40 0 40 0 0 -40 0 -40 40 0 40 0 0 -40 0 -40 40 0 40 0 0 80 0 80 -40 0 -40 0 0 40 0 40 -40 0 -40 0 0 40 0 40 -40 0 -40 0 0 40 0 40 -40 0 -40 0 0 40 0 40 -40 0 -40 0 0 40 0 40 -80 0 -80 0 0 -40z M560 520 l0 -40 -40 0 -40 0 0 -40 0 -40 -40 0 -40 0 0 -40 0 -40 -40 0 -40 0 0 -40 0 -40 -40 0 -40 0 0 -40 0 -40 -40 0 -40 0 0 -40 0 -40 -40 0 -40 0 0 -40 0 -40 80 0 80 0 0 40 0 40 40 0 40 0 0 40 0 40 40 0 40 0 0 40 0 40 40 0 40 0 0 40 0 40 40 0 40 0 0 40 0 40 40 0 40 0 0 80 0 80 -40 0 -40 0 0 -40z"/></g></svg> C–OH ⇔ C=O + H^+^ + e^−^, *etc.*, at the electrode interfaces.^50,51^ Certainly, the detailed electrochemical reaction mechanism still need be further studied.

**Fig. 7:**
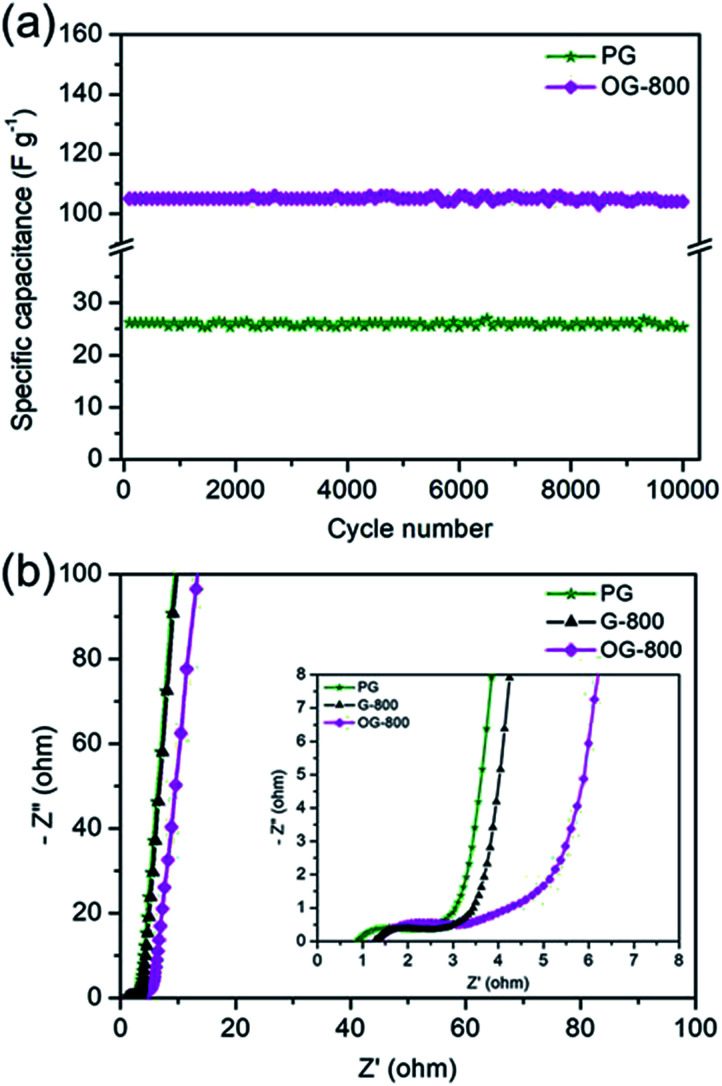
(a) The cycling profile of PG and OG-800 measured at a current density of 2 A g^−1^. (b) Nyquist plots of PG, G-800 and OG-800 electrodes, the inset shows the enlarged EIS at high frequency region.

The cycling stability was tested by galvanostatic charge/discharge at a current density of 2 A g^−1^. Fig. 7a shows the cycle durability of PG and OG-800 over 10 000 charge–discharge cycles. OG-800 shows a retention rate of 98% of its initial capacitance, demonstrating excellent electrochemical stability. Although, the specific capacitance of PG is low, its retention rate is also as high as 96.8%. In addition, Nyquist plots of PG, G-800 and OG-800 are shown in Fig. 7b. A sharp increase at low frequencies indicates the capacitive behavior of the electrodes. The slope of the 45° portion of the curves is the Warburg resistance and is a result of the frequency dependence of ion diffusion in the electrolyte to the electrode interface.^52^ Moreover, the semicircles are mainly related to the summation of the contact resistance and charge transfer resistance. The equivalent series resistance (ESR) value determined from the first intersection of the semicircle with the real axis.^31,52^ The ESR of PG, G-800 and OG-800 are 0.89, 1.29 and 1.34 Ω, respectively. The small semicircle diameter and ESR observed for all three electrodes, which match well with their outstanding rate performance.

## Conclusions

In summary, graphene sheets with different content of oxygen functional groups have been prepared through thermal reduction of GO, and their electrochemical performances were investigated as electrode materials for supercapacitors. Moreover, the electrochemical performance of G-800, OG-800 and PG were compared. The structure and surface properties, especially the effect of oxygen functional groups on the specific capacitance of graphene electrodes were explored systematically. The content and species of oxygen functional groups were proved to be critical factors influencing the capacitive performance of graphene electrochemical supercapacitors. The specific capacitance decreases when the oxygenated species reduces. In addition, at 20 mV s^−1^, the specific capacitance of G-800 can be increased from 23 to 107 F g^−1^ after nitric acid oxidation treatment. However, the specific capacitance of PG is only 27 F g^−1^. These results have special references to design and prepare graphene-based supercapacitor electrode materials.

## Conflicts of interest

There are no conflicts to declare.

## Supplementary Material

RA-008-C7RA12425B-s001
