# Supplementary material for: Oxygen functional groups improve the energy storage performances of graphene electrochemical supercapacitors
Source: RSC Adv. 2018 Jan 12;8(6):2858–65. doi: 10.1039/c7ra12425b (PMC9077562; doi:10.1039/c7ra12425b)
Supplement: RA-008-C7RA12425B-s001 [file RA-008-C7RA12425B-s001.pdf]

Electronic Supplementary Material (ESI) for RSC Advances.
This journal is © The Royal Society of Chemistry 2018

Supporting Information for

Oxygen functional groups improve the energy storage performances of graphene electrochemical supercapacitors

Hailiang Cao,*[,a,b] Xing Peng,[a] Min Zhao,[a] Peizhi Liu,[a] Bingshe Xu [a] and Junjie Guo*[,a]

a Key Laboratory of Interface Science and Engineering in Advanced Materials, Ministry of Education, Research Center of Advanced Materials Science and Technology, Taiyuan University of Technology, Taiyuan, 030024, China.

b Key Laboratory of Graphene Technologies and Applications of Zhejiang Province, Advanced Li-ion Battery Engineering Lab, Ningbo Institute of Materials Technology and Engineering (NIMTE), Chinese Academy of Sciences, Ningbo 315201, China.

**Corresponding author: E-mail: caohltyut@sohu.com; guojunjie@ tyut.edu.cn*

Fax: +86-531-6010-236; Tel: +86-531-6010-236

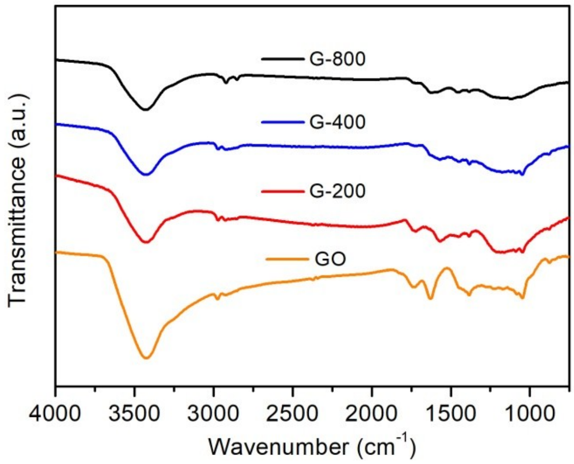

Fig. S1 FTIR transmittance spectra of GO, G-200, G-400 and G-800.

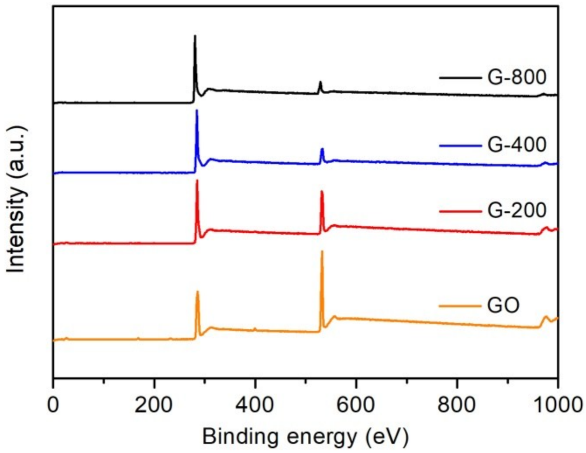

Fig. S2 XPS spectra of GO, G-200, G-400 and G-800.

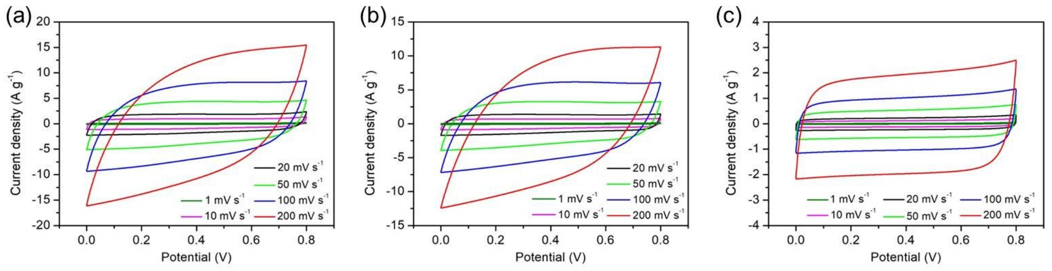

Fig. S3 CV curves of G-200 (a), G-400 (b) and G-800 (c) electrodes at different scan rates within a potential range of 0-0.8 V in 5 M KOH.

.

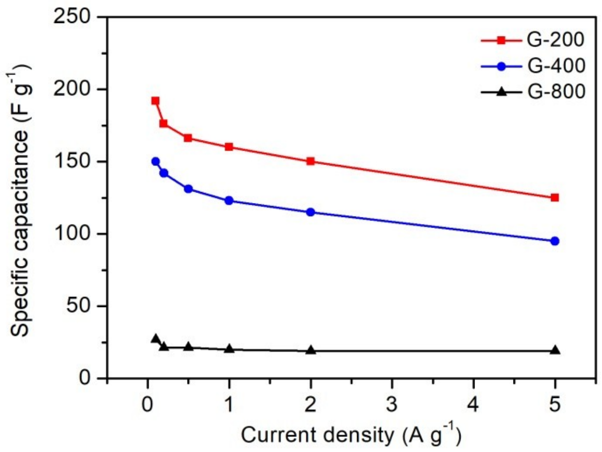

Fig. S4 Specific capacitance of G-200, G-400 and G-800 at different current densities.

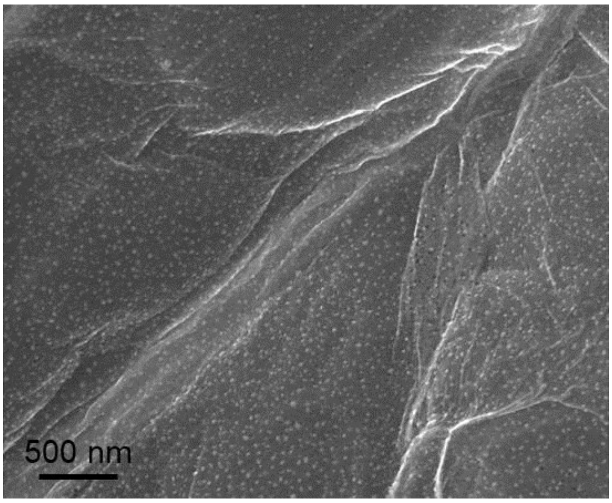

Fig. S5 SEM image of graphene-Co composite after thermal treatment at 800 °C.

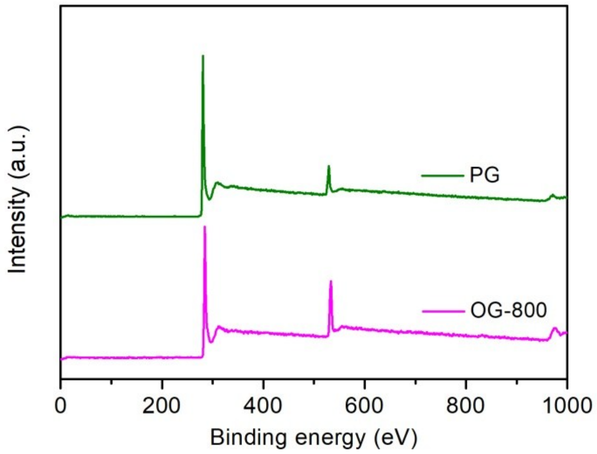

Fig. S6 XPS spectra of PG and OG-800.

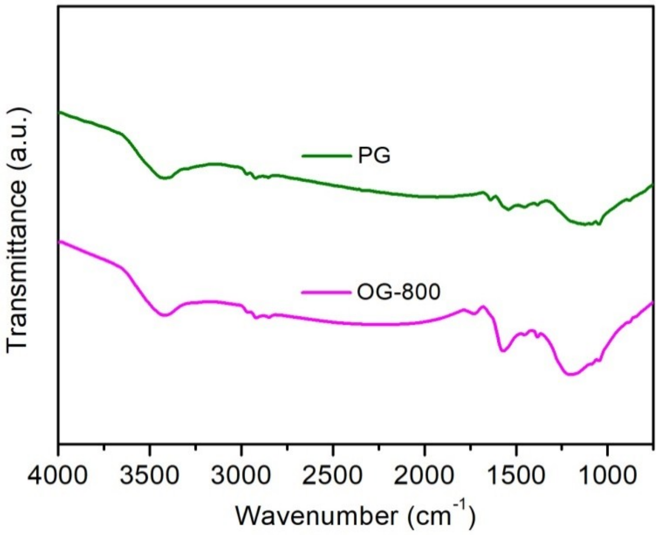

Fig. S7 FTIR transmittance spectra of OG-800 and PG.

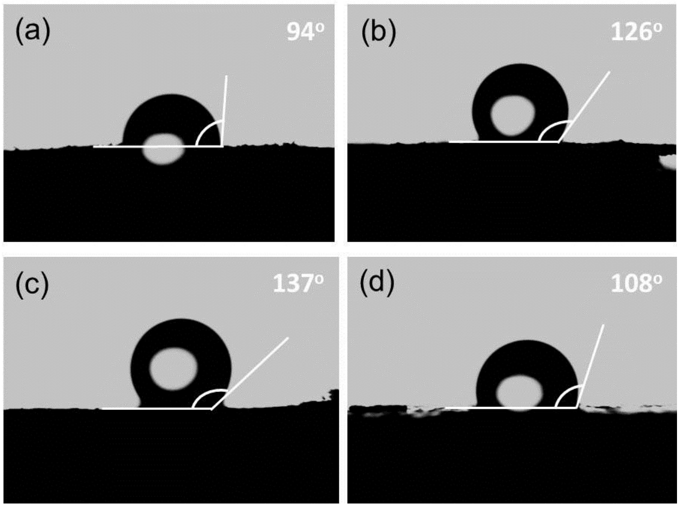

Fig. S8 Contact angle measurements of G-200 (a), G-400 (b), G-800 (c) and OG-800 (d).

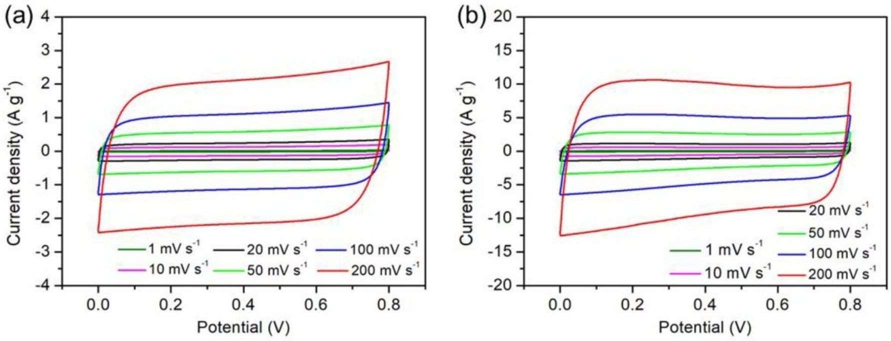

Fig. S9 CV curves of PG (a) and OG-800 (b) electrodes at different scan rates within a potential range of 0-0.8 V in 5 M KOH.

Table S1. Specific surface area, I_D/I_G, XPS elemental analysis (at. %), XPS data of C1s peaks: binding energies and relative area percentages (in parentheses) and specific capacitance of samples.

Sample	G-200	G-400	G-800	PG	OG-800
BET surface area (m^2 g^{-1})	248	302	251	116	240
I_D/I_G	0.96	0.94	0.93	1.04	0.92
C	80.4	89.3	93.1	93.4	86.6
O	19.6	10.7	6.9	6.6	13.4
C/O	4.1	8.3	13.5	14.2	6.5
C-C	284.6 (56.55)	284.6 (59.80)	284.6 (63.15)	284.6 (63.23)	284.6 (60.03)
C-OH	285.8 (16.63)	285.6 (17.89)	285.7 (15.82)	285.8 (15.20)	285.5 (13.17)
C=O	287.0 (10.9)	286.9 (9.40)	286.8 (10.02)	286.9 (8.45)	286.8 (11.62)
COOH	289.0 (15.91)	289.5 (12.91)	290.0 (11.01)	289.8 (13.12)	289.0 (15.18)
Capacity (F g^{-1})					
20 mV s^{-1}	174	127	23	27	107
